# Inaugural annual special section of the intellectual and developmental disabilities research centers: developmental cognitive neuroscience and neurodevelopmental disorders

**DOI:** 10.1186/s11689-018-9258-5

**Published:** 2018-12-13

**Authors:** Shafali Spurling Jeste, Charles A. Nelson

**Affiliations:** 1 Psychiatry, Neurology, Pediatrics, UCLA David Geffen School of Medicine, UCLA Center for Autism Research and Treatment, Los Angeles, USA; 2 Pediatrics, Harvard Medical School, Boston Children’s Hospital, Boston, USA

As a child neurologist (SSJ) and developmental cognitive neuroscientist (CAN), we care for and study individuals across a wide developmental, age, and ability spectrum. Over the years, as we have engaged with and learned from families of individuals with a variety of neurodevelopmental disorders, including intellectual and developmental disabilities (IDDs), we have been struck by the impact of the rapid advances that have been made in the field over the last several decades. Parents of adults with IDD often report that years ago, when they were first concerned about their child’s development (i.e., due to motor delays, early seizures, or failure to gain language) few, if any educational, medical, or therapeutic resources were available for their children. Many were told by their health care provider not to worry because their child would “eventually catch up,” while others were instructed to just “watch and wait, because [boys] develop more slowly.” Those who eventually received a genetic diagnosis, usually in late childhood, received little to no concrete information about the functional significance (including treatment options) of the genetic variant or mutation, thus perpetuating continued uncertainty and fear about their child’s future. In contrast, parents with recently diagnosed infants and children often share stories of hope and empowerment, with early detection of developmental delays facilitating prompt intervention and genetic testing which, in turn, leads to improved clinical monitoring and prognostication, engagement with patient advocacy groups, and new opportunities for entry into patient registries, natural history studies, and clinical trials.

Improved screening, clinical care, treatment, and advocacy for individuals with IDDs result directly from decades of collaborative, multidisciplinary research that was first formally supported through the Mental Retardation Facilities and Community Health Centers Construction Act (Public Law 88-164). Signed into law by John F. Kennedy in 1963, this Act drastically altered the delivery of health and education services to those with IDDs. Prior to this legislation, individuals with intellectual disability or other neurodevelopmental disorders were institutionalized or hospitalized rather than rehabilitated and integrated. This Act began a system of special education and community service programs for children and adults with IDDs. It also provided critical funding to establish national research centers, now known as the Eunice Kennedy Shriver Intellectual and Developmental Disabilities Research Centers (IDDRCs). For more than 50 years, these centers, funded by the *Eunice Kennedy Shriver* National Institute of Child Health and Development (NICHD), have catalyzed multidisciplinary research in IDDs, from molecular biology, developmental neuroscience, and neurogenetics to clinical studies focused on developmentally informed assessment and treatment of individuals with IDDs. Each center contains core facilities and projects that support research in IDDs and that train the next generation of both basic and clinical investigators. While the clinical and/or scientific cores vary across sites, including areas such as molecular genetics, bioinformatics, cellular imaging, and preclinical models, every IDDRC is required to include a clinical translational core to promote patient-centered research.

Unified by the theme of Developmental Cognitive Neuroscience, this special issue of the *Journal of Neurodevelopmental Disorders* celebrates the breadth, innovation, collaboration, and creativity of these centers, with contributions from Children’s Hospital of Philadelphia, Children’s National Medical Center, Kennedy Krieger Institute, University of California Los Angeles, University of North Carolina, Chapel Hill, Vanderbilt University and Washington University. These authors represent a range of professional backgrounds, from child neurology and psychiatry to neuroscience and developmental psychology, and the articles cover a diversity of topics, including innovations in neuroimaging of infants at high risk for autism [1], arterial spin labeling in autism [2], sex differences in attention-deficit hyperactivity disorder [3], functional imaging of memory systems [4], quantitative assessments of motor function in children [5], mechanisms underlying language learning [6], neural networks implicated in learning disabilities [7], and neurodevelopment in preterm infants [8]. Through the field of developmental cognitive neuroscience, we have learned about the functional and structural neuroanatomy and aberrant neural circuitry of various neurodevelopmental disorders. These scientific insights are illustrated in several of the papers in this special issue that introduce and explore research on the developmental underpinnings of both typical and atypical neurodevelopment, providing insight on mechanisms underlying IDDs and introducing new methods to study the developing brain.

This inaugural annual issue featuring work of the IDDRCs showcases research in Developmental Cognitive Neuroscience in individuals with a range of IDDs as well as typical development, illustrating the breadth and depth of cutting-edge, interdisciplinary science taking place across these centers. It has become increasingly clear in the field of neurodevelopmental disorders that only through partnerships will true innovation occur. We can consider the example of tuberous sclerosis complex (TSC), a genetic syndrome highly penetrant for IDD as well as epilepsy. Breakthroughs in molecular genetics and neurobiology, many of which were spearheaded by investigators in the Harvard-Boston Children’s Hospital IDDRC, elucidated the specific molecular pathway, mediated by mTOR (Mammalian Target of Rapamycin), that leads to the neurodevelopmental features of this condition. Identification of this potential therapeutic target has prompted several preclinical studies and now clinical trials of mTOR inhibition for various neurological sequelae of TSC, such as epilepsy, intracranial hamartomas, and cognitive impairment. It also was recognized clinically that these children often are diagnosed in utero, through prenatal ultrasound, motivating prospective studies of early development and prediction of IDD in infancy. Through a multisite collaboration between UCLA and Harvard, we identified features of atypical development in the first year of life that predict autism and cognitive impairment [9, 10], and these findings have led to the first randomized controlled clinical trial of early intervention for infants with TSC (JETS: JASPER Early Intervention for TSC; NCT03422367). In parallel, prospective studies of epilepsy in TSC have identified electrophysiological predictors of seizures in the first year of life, leading to the first clinical trial of epilepsy prevention in these infants (PREVeNT: Preventing Epilepsy Using Vigabatrin in Infants with TSC; NCT02849457). Each of these trials has developed strategies to ensure that geographically and socioeconomically diverse families are able to participate in these studies, including delivery of the intervention remotely (JETS) and financial support for travel (PREVeNT).

Ten years ago, a TSC diagnosis would have been coupled mostly with counseling about the high risk for epilepsy, autism, and IDD, with variable intensity of monitoring for the development of these conditions. Now, through scientific advances, a TSC diagnosis is accompanied by concrete steps towards therapeutics, with the goal of modifying disease course and maximizing neurodevelopmental gains in these children.

As far as we have come, we also recognize that we are just approaching the precipice of an era in which technological advances, illustrated by the articles on Developmental Cognitive Neuroscience included in this special issue, can greatly accelerate our efforts to improve outcomes in individuals with IDDs. With improved precision and accessibility of clinical genetic testing, we have identified thousands of genetic etiologies of IDDs. Each of these rare disorders alone accounts for less than 1% of IDDs, but taken together, they account for more than 15%. Given the molecular, neurobiological and even clinical convergence of many of these rare variants, we need mechanisms for IDDRC-wide patient registries that are easily accessible and integrated with the electronic medical record. We also need more scalable and systematic protocols for clinical phenotyping. These efforts will accelerate our understanding of these conditions and will help identify the most robust clinical endpoints for trials. Additionally, given the rapid advances in functional and structural imaging of the developing brain, we are identifying meaningful functional biomarkers that can shed light on predictors of atypical development in early infancy, can elucidate mechanisms underlying the genetic and molecular disruptions that cause IDDs, and ultimately, can serve as functional biomarkers of drug target engagement and surrogate outcomes in clinical trials. We also must continue to encourage more integrative intervention studies, in which targeted pharmacotherapy is coupled with behavioral intervention, with more creative methods for remote assessment, monitoring and intervention delivery to maximize inclusion of families in under-resourced areas.

Last year, one of us (SSJ) gave a lecture at a family meeting for a genetic syndrome defined by a micro-duplication on chromosome 15q. This chromosomal variant, called Dup15q Syndrome, is one of the most common copy number variants associated with IDDs. The meeting, sponsored by the patient advocacy group, the Dup15q Alliance, assembled patients, advocates, clinicians, and researchers to discuss current topics in both the clinical care and scientific discoveries in this syndrome. As I presented the advances we had made in the discovery of a functional biomarker and its implications for a potential clinical trial, I felt confident and proud of the accomplishments, assuming that the rest of the audience, mostly parents of affected individuals, would be equally enthused. After the talk, an adult with Dup15q syndrome approached the microphone with the help of his father, his spasticity and motor impairment evident in his slow and unsteady gait. He quietly and slowly, with limited articulation, asked the following question: “When will you find a cure? I have been waiting my whole life for one.” I was immediately reminded that we must remain humbled by the daily challenges that individuals with IDDs face, while communicating our discoveries in a way that infuses hope and inspiration. With this first issue of JNDD portraying some of the innovative work of IDDRC investigators, our ultimate goal is to conduct research that will result in meaningful improvements in the wellbeing of these individuals and their families.
